# Early-Stage Non-Small Cell Lung Cancer: Prevalence of Actionable Alterations in a Monocentric Consecutive Cohort

**DOI:** 10.3390/cancers16071410

**Published:** 2024-04-03

**Authors:** Rossella Bruno, Anello Marcello Poma, Martina Panozzi, Alessandra Lenzini, Gianmarco Elia, Carmelina Cristina Zirafa, Vittorio Aprile, Marcello Carlo Ambrogi, Editta Baldini, Marco Lucchi, Franca Melfi, Antonio Chella, Andrea Sbrana, Greta Alì

**Affiliations:** 1 Unit of Pathological Anatomy, University Hospital of Pisa, Via Roma 67, 56126 Pisa, Italy; m.panozzi@studenti.unipi.it; 2 Department of Surgical, Medical, Molecular Pathology and Critical Area, University of Pisa, Via Savi 10, 56126 Pisa, Italy; marcello.poma@med.unipi.it (A.M.P.); a.lenzini2@studenti.unipi.it (A.L.); g.elia3@studenti.unipi.it (G.E.); vittorio.aprile@unipi.it (V.A.); marcello.ambrogi@unipi.it (M.C.A.); marco.lucchi@unipi.it (M.L.); greta.ali@unipi.it (G.A.); 3 Minimally Invasive and Robotic Thoracic Surgery, Department of Surgical, Medical, Molecular and Critical Care Pathology, University Hospital of Pisa, Via Paradisa 2, 56124 Pisa, Italy; carmelina.zirafa@ao-pisa.toscana.it (C.C.Z.); franca.melfi@unipi.it (F.M.); 4 Medical Oncology, Hospital of Lucca, 55100 Lucca, Italy; editta.baldini@uslnordovest.toscana.it; 5 Unit of Pneumology, University Hospital of Pisa, Via Roma 67, 56126 Pisa, Italy; anto.kell@tiscali.it (A.C.); andrea.sbrana@ao-pisa.toscana.it (A.S.)

**Keywords:** early-stage non-small cell lung cancer, targeted therapy, immunotherapy, actionable alterations, PD-L1, predictive biomarkers

## Abstract

**Simple Summary:**

The therapeutic scenario of early-stage (ES) non-small cell lung cancer (NSCLC) is rapidly evolving. Precision medicine, including both targeted therapy and immunotherapy, has recently entered the clinical practice of neoadjuvant and adjuvant settings. However, only a few data are available about oncogene addiction of ES tumors. In this study, we determined the prevalence of the main lung cancer actionable alterations in a consecutive monocentric cohort of ES-NSCLC. We found that the prevalence of targetable alterations was similar between ES and advanced NSCLC, with a significant enrichment in *MET* exon 14 skipping alterations in ES-NSCLC. Our results can support the role of a biomarker testing strategy to improve the management of ES lung cancer patients.

**Abstract:**

Early-stage (ES) non-small cell lung cancer (NSCLC) is diagnosed in about 30% of cases. The preferred treatment is surgery, but a significant proportion of patients experience recurrence. Neoadjuvant and adjuvant chemotherapy has a limited clinical benefit. EGFR tyrosine kinase inhibitors and immunotherapy have recently opened new therapeutic scenarios. However, only a few data are available about the ES-NSCLC molecular landscape and the impact of oncogene addiction on therapy definition. Here, we determined the prevalence of the main lung cancer driver alterations in a monocentric consecutive cohort. Molecular analysis was performed on 1122 cases, including 368 ES and 754 advanced NSCLC. The prevalence of actionable alterations was similar between early and advanced stages. ES-NSCLC was significantly enriched for *MET* exon-14 skipping alterations and presented a lower prevalence of *BRAF* p.(V600E) mutation. PD-L1 expression levels, evaluated according to actionable alterations, were higher in advanced than early tumors harboring *EGFR*, *KRAS*, *MET* alterations and gene fusions. Taken together, these results confirm the value of biomarker testing in ES-NSCLC. Although approved targeted therapies for ES-NSCLC are still limited, the identification of actionable alterations could improve patients’ selection for immunotherapy, favoring the enrollment in clinical trials and allowing a faster treatment start at disease recurrence.

## 1. Introduction

Lung cancer is the second most common cancer and it is one of the main causes of cancer-related death worldwide. In the United States, it has been estimated that approximately 350 people die each day from lung cancer, and 81% of cases are related to cigarette smoking [1]. In Europe, lung cancer accounts for 20% of total deaths [2].

Overall, about 80–85% of cases consist of non-small cell histology and adenocarcinoma is the most common histotype [1]. The majority of non-small cell lung cancer (NSCLC) cases are diagnosed in an advanced or metastatic stage, associated with poor prognosis, with a 5-year survival rate of about 4–5% [3]. Advancement in our knowledge of genomic landscape of lung cancer has greatly changed and improved the therapeutic scenario, leading to the era of precision medicine, including both targeted therapies and immunotherapy. In particular, a significant proportion of NSCLCs harbor actionable mutations in oncogenes able to drive cancer development and progression. Some of these actionable alterations can be targeted by approved drugs [4] and mainly fall within the following oncogenes: epidermal growth factor receptor (*EGFR*), Kirsten rat sarcoma virus mutations (*KRAS*), anaplastic lymphoma kinase (*ALK*), B-Raf murine sarcoma viral oncogene homolog B (*BRAF*), ROS proto-oncogene 1 (*ROS1*), mesenchymal–epithelial transition factor (*MET*), human epidermal growth factor receptor-2 (*HER2*), rearranged during transfection (*RET*), and neurotrophic tyrosine receptor kinase 1, 2, and 3 (*NTRK1/2/3*). The frequency of oncogenic drivers in NSCLC can differ among populations and gender [5,6]. For instance, *EGFR* common actionable mutations have a prevalence of about 47% in Asians and about 16% in Caucasians [6]. Considering that oncogene-addicted (i.e., tumors with actionable alterations) advanced and metastatic NSCLC can greatly benefit from approved targeted therapies, the molecular testing of the aforementioned oncogenes is mandatory for this tumor setting [4,7].

Similarly to the advanced and metastatic stages, precision medicine has recently entered and improved the clinical management of early-stage (ES) NSCLC [8].

ES-NSCLC is diagnosed in about 30% of cases, referring to tumors with a pathological stage from I to IIIA, according to the American Joint Committee on Cancer (AJCC) tumor–nodes–metastases (TNM) classification [9]. Surgery is the main modality of treatment and, until recently, the post-operative standard of care was represented by a platinum-based two-drug combination chemotherapy [8]. However, long-term survival for surgically resected tumors remains poor, equal to 5.4% at 5 years, with an overall survival rate ranging from 90% to 12% in stages I and III, respectively [10,11,12]. To date, stage IB-IIIA resected NSCLC with *EGFR* exon 19 deletions or exon 21 p.(L858R) mutation can benefit from adjuvant treatment with osimertinib, a third-generation EGFR tyrosine kinase inhibitor (TKI). Adjuvant osimertinib has been approved by both the United States Food and Drug Administration (FDA) and the European Medicines Agency (EMA) on the basis of the results of the phase III randomized ADAURA trial [13,14]. The adjuvant treatment with osimertinib provided a significant overall survival (OS) benefit versus placebo. In the overall enrolled population, the 5-year survival rate was 88% in the osimertinib group and 78% in the placebo group, independent of tumor stage and with or without adjuvant chemotherapy [14]. Also, adjuvant atezolizumab, a Programmed Death Ligand-1 (PD-L1) inhibitor, entered the clinical practice of IB-IIIA resected ES-NSCLC. The phase III Impower010 study showed a benefit in disease-free survival (DFS) of adjuvant immunotherapy in comparison to chemotherapy alone [15]. In detail, an OS improvement in favor of atezolizumab was reported in the stage II-IIIA tumors with a PD-L1 tumor proportion score (TPS) > 1% and the benefit was greatest in the stage II-IIIA tumors with a PD-L1 TPS > 50%. FDA approved atezolizumab for the adjuvant treatment of stage II-IIIA tumors with PD-L1 expression levels greater than 1%, while EMA approved adjuvant atezolizumab for high-risk tumors with a PD-L1 expression greater than 50% and in the absence of *EGFR* mutations and *ALK* rearrangements [8,15,16]. Similarly, on the basis of the Keynote-091 study [17], a programmed cell death 1 (PD-1) inhibitor, pembrolizumab, has been approved for the adjuvant treatment of NSCLC stages IB-IIIA. Pembrolizumab significantly prolonged DFS versus placebo regardless of PD-L1 status and genomic tumor testing [17,18].

In the neoadjuvant setting, the CheckMate 816 trial led to the FDA and EMA approval of nivolumab plus platinum-based chemotherapy for patients with stage IB-IIIA NSCLC and a PD-L1 expression level greater than 1% [19,20]. In fact, this combination provided a greater event-free survival benefit in patients with a tumor PD-L1 expression level of 1% or more than in those with a level of less than 1%. Nivolumab plus chemotherapy showed a benefit in terms of both pathological complete response and major response in comparison to chemotherapy alone [21].

In addition, different clinical trials with specific TKIs, already approved in the metastatic setting, and immune checkpoint inhibitors (ICIs) are currently ongoing for neoadjuvant and adjuvant treatment of ES-NSCLC [22,23]. Considering that lung cancer surveillance programs are growing wider in clinical practice, more patients are expected to be diagnosed with ES-NSCLC in the coming years [24]. However, only a few data are available about the molecular landscape of ES tumors, including the prevalence and the impact of oncogene addiction, different from *EGFR*, on therapy definition and prognosis. For instance, the benefit of immunotherapy in ES tumors harboring actionable oncogene mutations besides *EGFR* and *ALK* alterations has not been evaluated [16,18,21,22]. In the metastatic setting, oncogene-addicted tumors, except for *KRAS*, do not benefit from immunotherapy [22]. Hence, it could be reasonable to also extend the complete molecular characterization to ES-NSCLC in order to better define therapy. To the best of our knowledge, only Muthusamy et al. demonstrated the value, in terms of cost-effectiveness, of multigene testing in resected early-stage lung adenocarcinoma in relation to immunotherapy administration. Indeed, they used a real-world clinic–genomic database and reported a prevalence of oncogene-addicted tumors, likely not responsive to ICI, similar to advanced stages [24].

In this context, the aim of our study was to determine and compare the prevalence of the main lung cancer driver alterations in a consecutive monocentric cohort of early- and advanced-stage lung adenocarcinomas. Our findings can further support the usefulness of a biomarker testing strategy for ES tumors, thus driving the definition of new treatment strategies.

## 2. Materials and Methods

### 2.1. Patients and Tumor Specimens

In this study, 1122 cases of NSCLC diagnosed at the University Hospital of Pisa from January 2020 to December 2022 were evaluated. Histological and cytological diagnoses were performed and reviewed by expert pathologists according to the WHO 2021 histological and immunohistochemical criteria [25]. Adenocarcinoma (ADC), adenosquamous carcinoma (ADC-SCC), and NSCLC not otherwise specified (NOS) were included in this study (Table 1). Tumors were classified as early or advanced stages on the basis of the latest AJCC-TNM classification [9]. Molecular analyses were executed on formalin-fixed paraffin-embedded (FFPE) samples from surgical resections, biopsies, and cell-blocks or on Papanicolaou stained smears. For each case, the most representative specimen, in terms of percentage of neoplastic cells, was selected to determine the status of predictive biomarkers routinely tested for advanced NSCLC [4,7]. Histopathological and clinical data were also reviewed and collected for each patient.

This study was conducted in accordance with the principles of the 1975 Helsinki Declaration and was approved by the local Ethics Committee. Written informed consent was obtained from patients before tumor biopsy or surgical resection. All cases were anonymized for this study and no sensitive data were used. This study did not interfere with routine clinical practice.

### 2.2. Gene Mutations

For each FFPE sample, DNA was purified from three 10 μm thick unstained sections after standard deparaffinization in xylene and rehydration in graded solutions of ethanol, while for cytologic smears, one stained slide was placed in xylene for 48 h to remove the coverslip; then, the slide was rehydrated in graded solutions of ethanol (99%, 95%, 70%, and 50%) for 10 min each.

All samples were enriched for cancer cells by manual macrodissection; DNA was then purified by the QIAamp DNA Mini Kit (Qiagen, Hilden, Germany) according to the manufacturer’s protocol. DNA concentration and fragmentation were determined using both a spectrophotometer and a real-time PCR kit (Diatech Pharmacogenetics, Jesi, Italy).

Briefly, clinically relevant gene mutations within *EGFR*, *BRAF*, *KRAS*, *MET* exon 14 skipping, and gene rearrangements involving *ALK*, *ROS1*, *RET,* and *NTRK 1/2/3* were analyzed.

Molecular tests were performed by different technologies, according to changing practice patterns over time. For samples collected in the year 2020, mutational analysis of *EGFR*, *KRAS,* and *BRAF* was executed by MALDI-TOF technology on a Sequenom platform (Agena Bioscience, San Diego, CA, USA), using the kit Myriapod Lung Status (Diatech Pharmacogenetics) according to manufacturer’s instructions [26], while *MET* exon 14 skipping alterations were determined by direct Sanger Sequencing [27]. For samples collected from 2021 to 2022, molecular analysis was performed by next-generation sequencing (NGS). In detail, the NGS amplicon-based panel Myriapod-NGS Cancer panel DNA (Diatech Pharmacogenetics, Jesi, Italy) was used according to the manufacturer’s protocol. This panel covered clinically relevant regions within 17 oncogenes including *EGFR*, *KRAS*, *BRAF*, *MET.* Sequencing was carried out on the MiSeq platform according to the manufacturer’s protocol (Illumina, San Diego, CA, USA).

### 2.3. Gene Fusions and PD-L1

Immunohistochemistry (IHC) was used to determine PD-L1, ALK, ROS1, and NTRK1/2/3 expression levels. A representative tissue block or cell-block from each lesion was selected. Tissue sections of 4 μm thickness were deparaffinized in xylene, rehydrated using a graded series of ethanol solutions, and then subjected to immunohistochemical staining. The following antibodies were used: rabbit monoclonal primary anti-ALK antibody (clone D5F3, Roche-Ventana), rabbit monoclonal primary anti-ROS1 antibody (clone D4D6, Cell Signaling Technology (CST), Danvers, MA), and rabbit monoclonal antibody anti-pan-TRK (clone EPR17341, Roche-Ventana). PD-L1 expression was evaluated using the rabbit monoclonal antibody anti-PD-L1 (clone SP263, Roche-Ventana) with the OptiView DAB IHC Detection Kit and OptiView Amplification Kit (Roche-Ventana). Immunostaining was performed as a fully automated assay using BenchMark XT automated slide stainers (Roche-Ventana). Negative controls were carried out by omitting the primary antibodies. In all cases, the immunohistochemical evaluation was performed independently by two pathologists who were blind to the clinicopathological characteristics and molecular data of the patients, as previously described [28,29,30,31,32].

Fluorescence in situ hybridization (FISH) was used to evaluate *RET* rearrangements, to confirm ROS1- and NTRK-positive IHC tests and to evaluate *ALK* rearrangements in case of equivocal IHC results. The following probes were used: Vysis LSI ALK Dual Color, Break Apart Rearrangement Probe (Abbott Molecular), Vysis 6q22 ROS1 Break Apart FISH Probe (Abbott Molecular), Vysis and LSI (1q23) NTRK1 Break Apart FISH Probe Kit (Abbott Molecular), Vysis (10q11) RET Break Apart FISH Probe Kit (Abbott Molecular).

Details about IHC and FISH tests are reported in Supplementary Materials.

### 2.4. Analysis of TCGA Data

Molecular and clinical data of the lung adenocarcinoma (LUAD) cohort of TCGA PanCancer Atlas were downloaded from cBioPortal (https://www.cbioportal.org/, accessed on 18 March 2024). Clinical information and data on gene mutations and fusions were matched by tumor sample barcode. Cases with pathologic tumor stage IIIB, IV, or not reported were excluded. The prevalence of targetable alterations (i.e., *BRAF* p.(V600E) mutation, *EGFR* activating mutations, *MET* exon 14 skipping mutations, *ALK*, *NTRK*1/2/3, *RET* and *ROS1* fusions and *KRAS* mutations (codons 12, 13, and 61)) was computed.

### 2.5. Statistical Analysis

Kruskal–Wallis test followed by Dunn’s test for multiple comparisons was used to assess differences in terms of PD-L1; a *p*-value of 0.05 was deemed significant. For categorical variables, a chi-square test with analysis of residuals was carried out; cells with standardized residuals above 2 in absolute value were considered significant. All analyses and plots were generated in R (v.4.3.2, https://www.r-project.org/, last accessed 15 December 2023).

## 3. Results

### 3.1. Study Population

Patients’ clinicopathological characteristics are summarized in Table 1. No significant differences between early- and advanced-stage tumors were observed in terms of age, sex, and histological tumor type. For ES tumors, molecular analysis was performed on surgical specimens in almost all cases.

### 3.2. Gene Alterations

**Figure 1:**
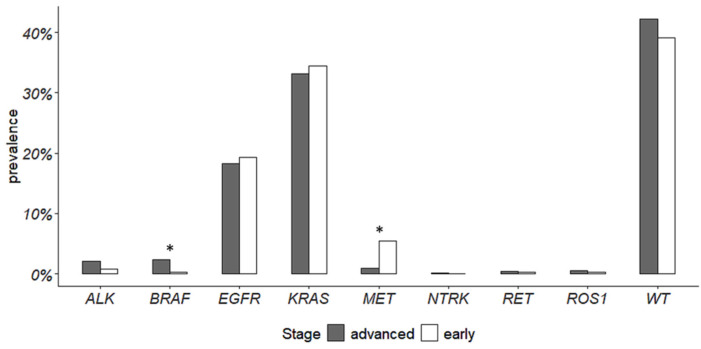
Prevalence of actionable gene alterations in advanced- and early-stage NSCLC. Asterisks indicate a significant difference.

The prevalence of the analyzed gene mutations and rearrangements was different between advanced- and early-stage NSCLC (Figure 1) (*p* = 0.005). Briefly, *BRAF* p.(V600E) and *MET* exon 14 skipping alterations significantly differed among the two groups. *BRAF* p.(V600E) alteration was more prevalent in advanced NSCLC, whereas *MET* exon 14 skipping alterations had a higher prevalence in ES-NSCLC. The other alterations did not show significant differences. Details about the prevalence of actionable gene alterations are reported in Table 2.

**Figure 2:**
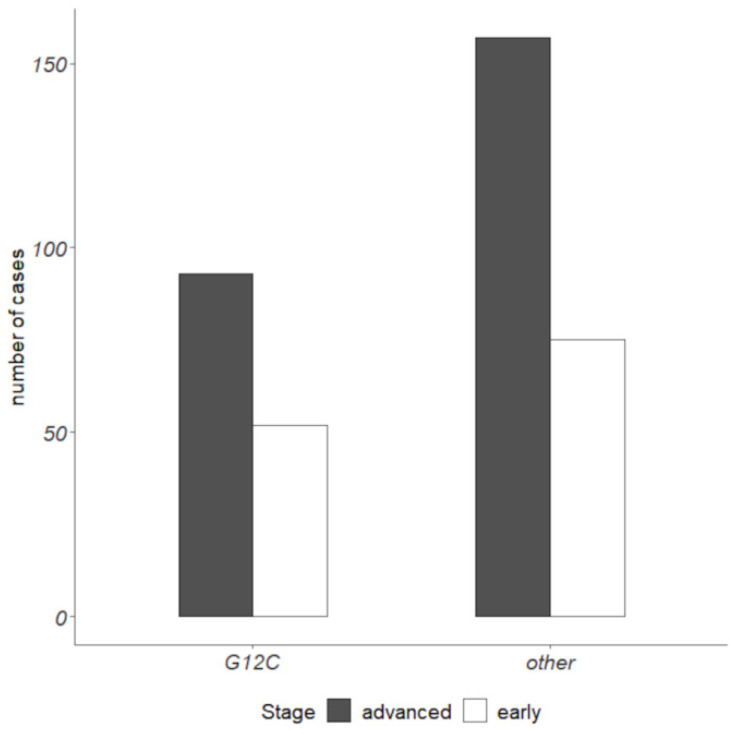
*KRAS* p.(G12C) prevalence in early-stage and advanced NSCLC cohorts.

The prevalence of the different types of *EGFR* mutations is reported in Table 3. Concerning *KRAS* mutations, in the ES-NSCLC setting, 40% of *KRAS* mutated cases harbored the p.(G12C) alteration, equal to 17% of all ES analyzed cases. In the advanced NSCLC setting, 37% of *KRAS* mutated cases had p.(G12C), equal to 14% of all advanced analyzed cases (Figure 2).

### 3.3. Prevalence of Actionable Alterations in Early-Stage Tumors of the LUAD TCGA Cohort

**Figure 3:**
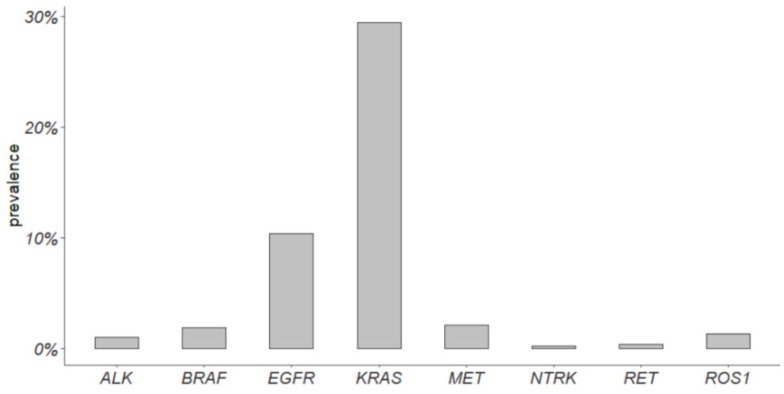
Prevalence of actionable gene alterations in early-stage NSCLC included in the LUAD TGCA cohort.

A total of 470 cases analyzed in the LUAD TCGA cohort were early-stage and were considered to compute the prevalence of actionable alterations (Figure 3). EGFR actionable alterations showed a prevalence of 10.5%, KRAS 29.4%, MET 2.1%, ALK 1%, RET 0.4%, ROS1 1.3%, and NTRK 0.2%. A comparison with advanced tumors was not performed due to the low number of stage IIIB and IV cases.

### 3.4. PD-L1 Expression Levels

**Figure 4:**
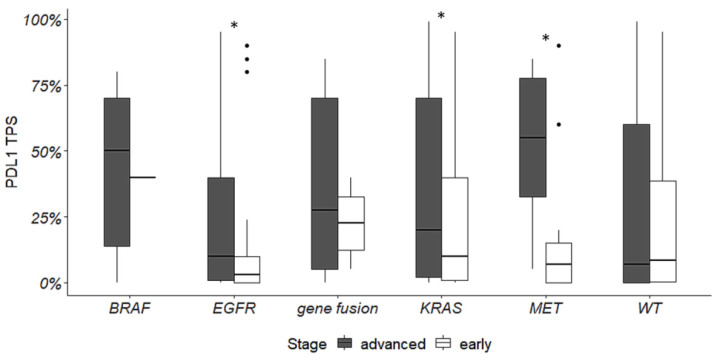
PD-L1 expression levels and actionable alterations in advanced and ES-NSCLC. Asterisks indicate a significant difference.

PD-L1 expression levels were evaluated in relation to actionable alterations both in ES and advanced NSCLC. PD-L1 expression was higher in advanced versus ES-NSCLC in the presence of *EGFR*, *KRAS,* and *MET* mutations (Figure 4).

In addition, we found that, in ES-NSCLC, PD-L1 expression levels were lower in *EGFR* mutated tumors (*p* = 0.02) compared to wild-type tumors, whereas no differences were observed in relation to other targetable alterations.

On the other hand, in advanced NSCLC, PD-L1 expression levels were higher in *BRAF* (*p* = 0.009), *KRAS* (*p* = 0.0001), *MET* (*p* = 0.02) mutated tumors and in tumors harboring gene fusions (*p* = 0.03) in comparison to wild-type tumors.

## 4. Discussion

ES-NSCLCs account for about 30% of lung cancer cases [9,24]. Surgery coupled with neoadjuvant or adjuvant chemotherapy, according to tumor stage and clinical characteristics, has been the standard of care for several years. However, disease recurrence is highly prevalent (30–50%), especially for resected stage III tumors [11,12]. Recently, the scenario of both neoadjuvant and adjuvant treatments of ES-NSCLC has changed with precision medicine entering clinical practice [8]. To date, evaluation of actionable alterations within *EGFR* and *ALK* genes and assessment of PD-L1 expression levels is part of the routine evaluation of ES-NSCLC [8,22], in order to select patients eligible for treatment with EGFR TKI [13] or immune checkpoint inhibitors [16,17].

Few data are available about the prevalence and the role of other rare targetable alterations in ES-NSCLC [24]. In this study, we confirmed, on a consecutive monocentric cohort, that the prevalence of the main driver alterations in ES and advanced NSCLC is comparable. Notably, we found significant differences for *MET* exon 14 skipping alterations, enriched in ES-NSCLC, and *BRAF* p.(V600E) mutation enriched in advanced NSCLC. Similar results have been previously reported by Muthusamy et al. in 2022. They have estimated the prevalence of actionable alterations in a cohort of 6697 NSCLCs, including 1177 early-stage tumors. In ES-NSCLC, they reported a prevalence of *EGFR* actionable alterations equal to 16.1%, *KRAS* 41.7%, *BRAF* p.(V600E) 1.5%, *MET* exon 14 skipping alterations 3.1%, *ALK* rearrangements 1.8%, *RET* 1.1%, *ROS1* 0.6%, and zero *NTRK*-positive cases. In comparison to our study, they found a significant enrichment only in *KRAS* mutations in ES-NSCLC and in *ALK* gene fusions in advanced NSCLC, but they reported a lower prevalence of *MET* exon14 skipping alterations (2.1%) and a higher prevalence of *BRAF* p.(V600E) (2.3%) in advanced tumors [24]. Overall, their findings are coherent with our results, even if in our cohort, neither *KRAS* mutations nor *ALK* fusions reached statistical significance (Figure 1). On the other hand, the prevalence of actionable alterations in early-stage tumors of the LUAD TGCA cohort differs both from our and Muthusamy’s studies. For instance, the prevalence of *EGFR* alterations is equal to 10.5% in ES LUAD TGCA cohort versus 20.3% and 16.1% in our and in Muthusamy’s study, respectively (Figure 3). These differences can be due to several reasons, the first of which is population characteristics, size, and data collection. Muthusamy et al. referred to a clinical–genomic database collecting data from hundreds of sites of care and their ES-NSCLC population mainly composed of Caucasian individuals also included other races (Asian, Black or African American, etc.) [24]. TCGA data are collected from many sites all over the world, different races are included, and datasets are extremely heterogeneous. Our data came from a single center and our study included only Caucasian patients, mainly from the same geographic area. It is well-known that the incidence of actionable alterations can differ among populations [6]. In addition, different molecular tests have been used in the aforementioned studies. Muthusamy et al. used a hybrid-capture NGS panel, and TCGA data came from whole exome sequencing, while we used both a hot-spot mass spectrometry assay and an amplicon-based NGS panel for gene mutations, and FISH and IHC for gene fusions. Moreover, TCGA data were obtained from freshly frozen tumor tissues, whereas both in our and in Muthusamy’s studies, FFPE tumor tissues were used, thus allowing tumor tissue enrichment. The type of molecular assay and biological material can impact on the detection of gene variants [33].

The higher prevalence of *MET* exon 14 skipping alterations in ES-NSCLC in comparison to advanced NSCLC deserves further investigation. In a previous study, Recondo et al. reported a prevalence of *MET* exon 14 skipping alterations equal to 2.8% in ES-NSCLC [34], while in our study, it was 5.4%. *MET* exon 14 skipping alterations are approved predictive biomarkers of response to TKI in the metastatic setting, but interesting case reports have already been published for ES-NSCLC as well. For instance, Fu and collaborators reported on a case of lung adenocarcinoma, stage IIIA-N2, positive for a *MET* exon 14 skipping alteration treated by savolitinib, a MET TKI, in the neoadjuvant setting. The patient achieved a pathological response equal to 50% and the final postoperative pathological staging was pT1cN0M0 [35]. In the same way, Wang and collaborators reported a case of a locally advanced NSCLC harboring a *MET* exon 14 skipping alteration who achieved a complete pathological response following neoadjuvant crizotinib. Interestingly, the described case developed rapid metastases due to discontinuation of short-term post-operative crizotinib, achieving a durable complete response after crizotinib rechallenge. This underlines not only the potential efficacy of neoadjuvant MET TKI, but also the importance of long-term postoperative targeted therapy [36].

Besides *MET*, a growing body of evidence is showing the feasibility of the neoadjuvant and adjuvant targeted therapies for early-stage lung cancer [22]. Osimertinib has been approved as adjuvant treatment for resectable *EGFR* mutant ES-NSCLC [7,8] and it is currently under evaluation in the neoadjuvant settings [37]. In fact, preliminary data have already demonstrated the safety and feasibility of neoadjuvant osimertinib for resectable *EGFR*-mutated NSCLC [38,39,40]. Then, completely resected ES-NSCLC harboring *ALK* rearrangements can benefit from an adjuvant treatment with alectinib, an ALK TKI, as revealed by the interim analysis of the ALINA trial. A significant DFS benefit was reported for alectinib versus chemotherapy, both in stage II-IIIA NSCLC and in the intention to treat populations [41]. The feasibility of alectinib has also been reported as a neoadjuvant treatment [42]. Regarding the efficacy of other targeted therapies in ES-NSCLC, conclusive data from clinical trials are still missing, owing to the rarity of some alteration types. However, evidence of the effectiveness of targeted therapies in oncogene-addicted ES-NSCLC is emerging from case reports. For instance, Goldman and collaborators have recently reported on a patient with a stage IB *KIF5B-RET* fusion-positive NSCLC achieving a complete pathological response on treatment with neoadjuvant selpercatinib, a RET TKI [43]. In the same way, Chen and collaborators described the case of a IIIA-NSCLC positive for a novel *LDLR-ROS1* fusion who received crizotinib as adjuvant treatment and achieved a recurrence-free survival of 29 months [44]. *BRAF* p.(V600E) mutation is rarer in ES than in advanced NSCLC. However, Liu et al. reported a major pathologic response in a patient with stage IIIA p.(V600E) lung adenocarcinoma who underwent a neoadjuvant-targeted therapy with BRAF and MEK inhibitors followed by radical surgical excision [45]. Available data, although preliminary, support the need for a more complete molecular characterization in daily practice.

In addition, despite recent approvals of ICI in both neoadjuvant and adjuvant settings, there are some concerns about their use in oncogene-addicted ES-NSCLC. Defining how and to what extent different molecular alterations may impact PD-L1 expression levels and immunotherapy response is crucial to better understand tumor genetic landscape and to improve patients’ clinical outcomes. Overall, it has been widely demonstrated that advanced disease may be more likely to express PD-L1 than earlier stages [46,47]. Furthermore, in our study cohorts, we found that PD-L1 expression levels were higher in advanced versus ES-NSCLC in the presence of *EGFR*, *KRAS,* and *MET* exon 14 skipping alterations. In comparison to wild-type tumors, in the ES-NSCLC cohort, PD-L1 expression levels were very low only in the presence of *EGFR* mutations, whereas, in advanced tumors, PD-L1 was greatly expressed in cases harboring *BRAF* p.(V600E), *KRAS,* and *MET* exon 14 skipping alterations and gene fusions. These results are all consistent with previous reports [47,48,49], supporting the strength of our findings. In the same way, Schoenfeld and collaborators, in a large cohort of lung adenocarcinoma, found that alterations in *KRAS* and *MET* were significantly associated with high expression of PD-L1, whereas *EGFR* mutations were associated with low PD-L1 levels. Genetic alterations directly impact the predictive value of PD-L1. In the metastatic setting, it has been reported that the clinical activity of ICI is lower in tumors with actionable driver alterations (except for *KRAS* mutations), though they can induce tumor regression in some cases. In this context, for patients with actionable alterations, targeted therapies and chemotherapy are recommended before considering immunotherapy [49]. In relation to ES-NSCLC, Su et al. evaluated the impact of driver mutations on the outcome of neoadjuvant treatment with ICI plus chemotherapy. Three out of eleven patients experienced disease recurrence; two of them harbored *EGFR* activating mutations (p.(L858R) and an exon 20 in frame insertion) and one had a *MET* exon 14 skipping alteration [50]. While the lower effectiveness of ICI in *EGFR* mutated tumors is well-known, it is not clear how the presence of *MET* exon 14 skipping alterations can impact immunotherapy response in both early and advanced NSCLC [51]. In the advanced setting, retrospective studies have demonstrated that tumors with *MET* exon 14 alterations can benefit more from immunotherapy than tumors with oncogene addictions other than *KRAS* mutations. Nevertheless, they still respond less than wild-type tumors [49,52,53]. In ES-NSCLC positive for *MET* exon 14 skipping, it might also be interesting to evaluate the effectiveness of combination therapies including ICI and TKI.

Overall, the decreased efficacy of ICI in oncogene-addicted tumors, even in the presence of high PD-L1 expression levels, is still under investigation. However, tumors with the most common actionable alterations usually present a low tumor mutational burden and a cold and immunosuppressive tumor microenvironment [54,55]. Consequently, it was hypothesized that, in these cases, PD-L1 expression may not depend on the interaction between immune and tumor cells, but it is more likely secondary to the activation of other oncogene pathways (e.g., JAK/STAT) [50,56]. Although limited evidence is still available, the identification of oncogene-addicted ES-NSCLC could guide clinical decisions in a more efficient and cost-effective manner. Avoiding ineffective immunotherapy in oncogene-addicted tumors could protect patients from unnecessary side effects and reduce the costs of treatment [24].

Besides the definition of neoadjuvant and adjuvant approaches, multigene testing of ES-NSCLC is also valuable for disease recurrence, which unfortunately is a common event. Patients with molecular results at diagnosis had less time between recurrence itself and the start of first-line treatment. This can have important consequences on the clinical outcome, but the impact of earlier molecular testing on the overall survival of ES-NSCLC has not yet been evaluated.

Current guidelines recommend broad molecular testing for advanced NSCLC, but increasing evidence demonstrates its value also in ES-NSCLC. Even if, at present, the only approved mandatory biomarkers for ES-NSCLC are *EGFR*, *ALK,* and PD-L1, the use of NGS panels rather than single gene tests is more cost-effective [4,24,33]. Considering that osimertinib is the only approved targeted therapy for ES-NSCLC, a complete molecular characterization of these tumors can be still hampered by National Health System reimbursement policies. In this scenario, it is necessary to promote clinical trials and multicenter studies in order to better define the therapeutic role of common and uncommon actionable oncogene alterations in ES-NSCLC.

In conclusion, in this study, we characterized a cohort of ES and advanced NSCLC, reporting a similar prevalence of actionable alterations. These results confirmed the value and convenience of multigene testing in ES-NSCLC, whose diagnostic–therapeutic algorithms are rapidly evolving. The identification of oncogene-addicted ES-NSCLC, which represents a considerable proportion of cases, is warranted to improve patients’ selection for immunotherapy, to facilitate patients’ enrollment in clinical trials on rare targetable alterations, and to better manage disease recurrence.

## 5. Conclusions

A biomarker testing strategy can improve the management of ES-NSCLC patients. Here, we confirmed that a considerable proportion of ES tumors harbored targetable alterations, similar to advanced stages. Furthermore, *MET* exon 14 skipping alterations are more prevalent in early than advanced stages, thus supporting further investigation on the efficacy of MET inhibitors in the neoadjuvant and adjuvant settings. Although current diagnostic algorithms of ES-NSCLC require only the evaluation of *EGFR* status, *ALK* fusions, and PD-L1 expression levels, the analysis of other rare actionable alterations can improve clinical practice. As already reported for the advanced setting, the presence of oncogene alterations, except for *KRAS* mutations, does not favor response to immune checkpoint inhibitors. Consequently, a complete molecular characterization of ES-NSCLC can be helpful to refine patients’ selection for immunotherapy. The identification of oncogene-addicted ES-NSCLC can also promote patients’ enrollment in clinical trials and improve the management of disease recurrence.

## Figures and Tables

**Table 1:** Clinicopathological features.

*Clinicopathological Features*	*All Patients* (N = 1122)	*Early-Stage* (N = 368)	*Advanced-Stage* N = 754)
***Age (years), median (IQR)***	71 (63–77)	72 (66–77)	71 (62–78)
***Sex, N (%)***			
*Female*	465 (41.4)	157 (42.7)	308 (40.8)
*Male*	657 (58.6)	211 (57.3)	446 (59.2)
***Histological diagnosis, N (%)***			
*ADC*	1017 (90.6)	366 (99.5)	651 (86.3)
*NSCLC NOS*	99 (8.9)	0	99 (13.2)
*ADCSCC*	6 (0.5)	2 (0.5)	4 (0.5)
***Materials, N (%)***			
*Cytology/cell-blocks*	253 (22.5)	0	253 (33.6)
*Biopsies*	410 (36.5)	3 (0.8)	407 (54)
*Surgical specimens*	459 (41)	365 (99.2)	94 (12.4)
***Tissue, N (%)***			
*Lung*	906 (80.7)	367 (99.7)	539 (71.5)
*Others*	216 (19.3)	1 (0.3)	215 (28.5)

ADC, adenocarcinoma; NSCLC NOS, non-small cell lung cancer not otherwise specified; ADC-SCC, adenosquamous cell carcinoma; IQR, interquartile range.

**Table 2:** Prevalence of actionable gene alterations.

Gene	Early-Stage NSCLC				Advanced-Stage NSCLC			
	Analyzed Cases	Wild Type	Actionable Alterations	Alteration Prevalence	Analyzed Cases	Wild Type	Actionable Alterations	Alteration Prevalence
***ALK fusions***	275	272	3	1.1%	646	630	16	2.5%
***BRAF p.(V600E)***	256	255	1	0.4%	503	486	17	3.4%
***EGFR*** *	349	278	71	20.3%	703	565	138	19.7%
***KRAS*** **	299	172	127	42.5%	672	422	250	37.2%
***MET exon 14 skipping***	241	228	13	5.4%	417	409	8	1.9%
***NTRK1/2/3 fusions***	82	82	0	0	156	155	1	0.6%
***RET fusions***	218	217	1	0.5%	434	431	3	0.7%
***ROS1 fusions***	255	254	1	0.4%	573	569	4	0.7%

* Molecular analysis involved actionable common and uncommon alterations within exons 18, 19, 20 and 21 of *EGFR* gene. ** Molecular analysis involved actionable alterations within codons 12, 13, and 61 of *KRAS* gene.

**Table 3:** Prevalence of *EGFR* mutations.

Early-Stage NSCLC				
*EGFR*	Number of Cases		Prevalence among *EGFR* Mutations	Prevalence among All Analyzed ES-NSCLC
Exon 19 in frame deletions	40		56.3%	11.5%
Exon 20 in frame insertions	7		9.9%	2%
p.(L858R)	16		22.5%	4.6%
Uncommon alterations	4		5.6%	1.1%
	*Mutation type*	*Number of cases*		
	*p.(G719A)*	2		
	*p.(L861Q)*	2		
Compound mutations	4			
	*Mutation type*	*Number of cases*	5.6%	**1.1%**
	p.(S768I) + p.(L858R)	1		
	p.(G719S) + p.(L861Q)	1		
	p.(E709Q) + p.(L858R)	1		
	p.(E709Q) + p.(G719C)	1		
**Advanced-stage NSCLC**				
***EGFR***	**Number of cases**		**Prevalence among *EGFR* mutations**	**Prevalence among all analyzed advanced NSCLC**
Exon 19 in frame deletions	70		50.7%	9.9%
Exon 20 in frame insertions	14		10.1%	2%
p.(L858R)	40		28.9%	56.9%
Uncommon alterations	4			
	*Mutation type*	*Number of cases*	2.9%	0.6%
	p.(G719C)	1		
	p.(G719A)	2		
	p.(S768I)	1		
*Compound mutations*	10			
	*Mutation type*	*Number of cases*	7.2%	1.4%
	p.(G719C) + p.(S768I)	1		
	p.(G719A) + p.(S768I)	1		
	p.(V689L) + p.(V744M) + p.(Y827F)	1		
	p.(E709A) + p.(G719S)	1		
	p.(E709A) + p.(G719C)	1		
	p.(S768I) + p.(L858R)	1		
	p.(A871G) + p.(L858R)	1		
	p.(S768I) + p.(V744M)	1		
	p.(774M) + p.(Y827F)	1		
	p.(S768M) + p.(V744M)	1		

## Data Availability

Data supporting the finding of this study are available from the authors on reasonable request.

## Supplementary Materials

The following supporting information can be downloaded at: https://www.mdpi.com/article/10.3390/cancers16071410/s1, details about immunohistochemistry and fluorescence in situ tests.
