# Supplementary material for: Early-Stage Non-Small Cell Lung Cancer: Prevalence of Actionable Alterations in a Monocentric Consecutive Cohort
Source: Cancers (Basel). 2024 Apr 3;16(7):1410. doi: 10.3390/cancers16071410 (PMC11010971; doi:10.3390/cancers16071410)
Supplement: Supplementary file 1 [file cancers-16-01410-s001.zip › cancers-2903242-supplementary.pdf]

Supplementary Materials

Immunohistochemistry

Tumor samples were considered positive for ALK when any percentage of tumor cells showed strong granular cytoplasmic staining. For ROS1, positive staining was characterized by diffuse cytoplasmic with some membranous staining of the tumor cells. The intensity of staining was graded as 0, no staining; 1+, weak staining; 2+, moderate staining in 1% to 50%; and 3+, strong cytoplasmic staining in more than 50% of tumor cells. With a result of 2+ or 3+, the confirmation of positivity was assessed by another orthogonal method. Regarding NTRK evaluation, any expression by IHC required confirmation by an orthogonal test. PD-L1 expression was assessed by TPS (Tumor Proportion Score) without considering staining intensity. The ratio between the number of positive neoplastic cells to the number of total neoplastic cells was calculated in three different areas and the average was considered. The result was deemed negative if the TPS was <1%, low expression if it was between 1% and 49%, and high expression if it was >50%. The minimum number of viable neoplastic cells to be examined was 100; when the number of cells was lower, the sample was considered insufficient. PD-L1 staining positivity was exclusively membrane positivity and does not consider its intensity; any mild to intense, complete, or partial membrane positivity was assessed and averaged over at least three different areas.

Fluorescence In Situ Hybridization

Fluorescence In Situ Hybridization (FISH) was performed on 4 to 6 μm thick paraffin sections of tumor tissues. Before hybridization, paraffin sections were deparaffinized in xylene (3 times, 10 minutes each), dehydrated by two 5-minute washes in 100% ethanol and two 5-minute washes in 96% ethanol, and air-dried at room temperature. Tissue sections were then transferred to a pretreatment solution at 80°C for 15 minutes, followed by a 3-minute wash in purified water, and incubated in a protease solution for 10 minutes at 37°C to digest proteins. After a brief washing in purified water, the slides were sequentially dehydrated in 70%, 85%, and 100% alcohol and air-dried at room temperature. Tissue sections were placed in a Hybrite (Abbott Molecular) for 3 minutes at 73°C to denature DNA, and probe hybridization was carried out overnight at 37°C. Tissue sections were washed in 0.1% NP40/2x SSC at 76°C for 4 minutes and then washed in 0.1% NP40/2x SSC at room temperature for 1 minute. Slides were mounted with 1.5 μg/mL 4',6-diamidino-2-phenylindole.

Tumor samples were scored by 2 independent investigators who were blind to the clinicopathological characteristics of the patients and to the immunohistochemical and molecular results. The test for *ALK* was considered positive if tumor cells had separate 5' (green) and 3' (red) probe signals or had isolated 3' signals. Regarding *ROS1*-rearrangement, two patterns were considered. One was the classic break-apart pattern, with 1 fusion signal (native ROS1) and 2 separated 3′ and 5′ signals. The other one, called atypical, considered an isolated 3′ (green) signal, with 1 fusion signal (native ROS1) and no 5′. All *ALK, ROS1, NTRK,* and *RET* FISH tests were considered positive if 15% or more of the tumor cells showed rearranged patterns of signals, whereas overlapping red and green signals (yellowish) indicated cells in which the genes were not rearranged.

For MET gene amplifications, according to Camidge criteria, a case was considered positive with a *MET*/CEP7 ratio > 2.2. The criteria for *HER2* amplification were as follows: *HER2*/CEP17 > 2 *HER2* is amplified, *HER2*/CEP17 < 2 but *HER2* copy number ≥ 6 *HER2* is amplified, *HER2* copy number < 4 *HER2* is not amplified, and *HER2* copy number is ≥ 4 but < 6 *HER2* amplification cannot be determined (*Sholl, L; Cooper, W; Kerr, K; Tan, D; Tsao, M; Jang, J "IASLC Atlas Of Molecular Testing For Targeted Therapy In Lung Cancer" 2023, Denver, Colorado*).
